# The vesicular transfer of CLIC1 from glioblastoma to microvascular endothelial cells requires TRPM7

**DOI:** 10.18632/oncotarget.26048

**Published:** 2018-09-07

**Authors:** Dominique Thuringer, Gaetan Chanteloup, Pascale Winckler, Carmen Garrido

**Affiliations:** ^1^ INSERM U1231, Laboratory of Excellence Ligue Nationale contre le Cancer, 21000 Dijon, France; ^2^ Université de Bourgogne Franche Comté, 21000 Dijon, France; ^3^ AgroSup Dijon, PAM UMR, DImaCell Imaging Facility, Université de Bourgogne Franche Comté, 21000 Dijon, France; ^4^ Centre Georges François Leclerc (CGFL), 21000 Dijon, France

**Keywords:** chloride intracellular channel, microRNA, exosome, transient receptor potential melastatin, glioblastoma

## Abstract

Chloride intracellular channel 1 (CLIC1) is highly expressed and secreted by human glioblastoma cells and cell lines such as U87, initiating cell migration and tumor growth. Here, we examined whether CLIC1 could be transferred to human primary microvascular endothelial cells (HMEC). We previously reported that the oncogenic microRNA, miR-5096, increased the release of extracellular vesicles (EVs) by which it increased its own transfer from U87 to surrounding cells. Thus, we also examined its effect on the CLIC1 transfer. In homotypic cultures, miR-5096 did not increase the expression of CLIC1 in U87 nor in HMEC. However, the endothelial CLIC1 level increased after exposure to EVs released by U87, and even more by miR-5096-loaded U87. The EVs-transferred CLIC1 was active in HMEC, promoting endothelial sprouting in matrigel. Cell exposure to EVs induced cytosolic Ca^2+^ spikes which were dependent on the transient receptor potential melastatin member 7 (TRPM7). TRPM7 silencing prevented Ca^2+^ spikes and the subsequent CLIC1 delivery into HMEC. Our data suggest that the vesicular transfer of CLIC1 between cells requires TRMP7 expression in recipient endothelial cells. How the vesicular transfer of CLIC1 is modulated in cancer therapy is a future challenge.

## INTRODUCTION

Extracellular vesicles (EVs) are membrane-enclosed particles released from either endosomes or the cell surface [1–3]. EVs are composed of an array of proteins, nucleic acids, lipids, and other metabolites that reflect the cell of origin. They offer an intercellular route to transfer oncogenic material that change the functions of non-malignant cells, i.e. proliferation, invasion, and angiogenesis [2]. Their secretion is correlated to the cell's ability to produce invadopodia (actin-rich cellular protrusions with proteolytic activity); i.e., inhibition of invadopodia formation decreased exosome release [3–5]. Importantly, glioblastoma (GBM)-derived EVs can cross the brain–blood-barrier and are detectable in the systemic blood circulation [6]. Profiling the composition of GBM-derived EVs may, therefore, offer a non-invasive means of assessing tumors *in situ* [4].

Studies have described extensive RNA expression analyses of GBM-derived EVs, however, proteomic profiles are currently limited [4, 7]. Among the vesicular proteins, one study identify the chloride intracellular channels (CLIC) carried by exosomes between GBM cells [8]. The CLIC family form a class of proteins that do not fit the paradigm set by classical ion channels (for review see; [9–11]). They can exist as both soluble globular proteins and integral membrane proteins with ion channel function. The first member of CLIC, namely CLIC1 (also known as NCC27), holds pathological implications in a variety of tumors, being involved in cell proliferation, motility, and angiogenesis [12–15]. CLIC1 is overexpressed in glioblastoma (GBM), with highest expression in patients with poor prognosis [13]. CLIC1 is also secreted in extracellular vesicles (EVs) by cancer cells [8] and is detected in biological fluids [8, 16, 17], fostering the hypothesis that secreted CLIC1 protein may increase GBM growth. Interestingly, Setti et al [8] have shown that the secretion of CLIC1 via EVs is common to all human GBM cell lines (U87MG, A172, LN405, U118MG, T98G, DBTRG-05MG and U373 MG). If the number of secreted EVs differs from one type of lineage to another, the membrane markers and biophysical properties of EVs are similar.

Using U87 GBM cell line, we have recently described that miR-5096 increases the outgrowth of filopodia in glioma cells, and promotes the extracellular release of EVs by U87 thereby promoting its own transfer to surrounding cells [18]. Here, we show that EVs also contain active CLIC1 whose amount is not significantly increased by miR-5096. The transfer of CLIC1 to human microvascular endothelial cells (HMEC) requires Ca^2+^ spikes and TRPM7 for their uptake, and contributes to endothelial sprouting [19, 20].

## RESULTS

### Extracellular vesicles from GBM cells transfer active CLIC1 to HMEC

**Figure 1:**
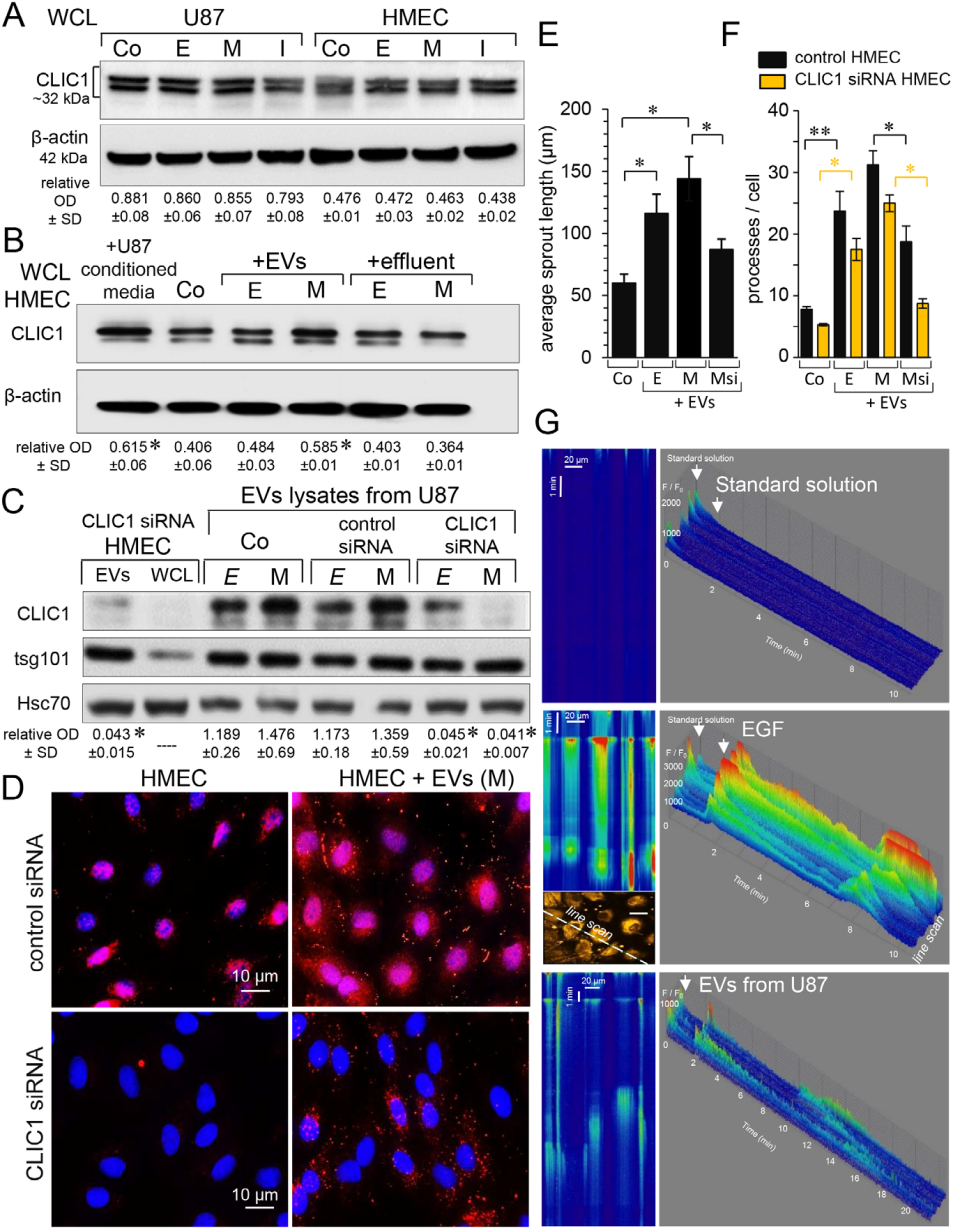
Active CLIC1 protein is transferred via vesicles from GBM to endothelial cells Immunoblot analysis of CLIC1 in whole cell lysates (WCL) from homotypic cultures of U87 and HMEC, 48 h after loading. Untreated cells were used as control (Co). Cells were loaded empty (E) or with 30nM miR5096 mimic (M) or inhibitor (I). β-actin as loading control (60μg proteins/lane). Numbers indicate mean values of optical densities (OD) of CLIC1 relative to β–actin (± SD; *P*>0.05 *vs* Co; n = 3). **(B)** CLIC1 increased in HMEC after 24 h of incubation with EVs. Cell-conditioned media were collected from homotypic U87 miR-loaded (M) or not (E), 48h after loading. HMEC were exposed to EVs or effluent (soluble fraction) separated from U87-conditioned media. Numbers indicate mean OD values of CLIC1 relative to β-actin (± SD; ^*^ *P*<0.05 *vs* Co; n = 3). **(C)** EVs contained CLIC1. Lysates of EVs were immunoblotted for the marker tsg101. Homotypic U87 were loaded (M) or not (E) upon transfection of control siRNA or siRNA targeting CLIC1. Silencing CLIC1 was also tested in HMEC (WCL) and HMEC-released EVs (Hsc70 as loading control). Numbers indicate mean OD values of CLIC1 related to tsg101 for EVs lysates (± SD; ^*^ *P*<0.05 *vs* Co (E); n = 4). **(D)** Endothelial cell localization of CLIC1. HMEC were silenced by siRNA CLIC1 then exposed to EVs collected from homotypic U87 (M) (n = 3). CLIC1 stained with alexa Fluor 594 (red) and nuclear DNA with Dapi (blue). **(E)** CLIC1 effect on the length of endothelial sprouts formed from spheroïds in Matrigel for 24h, in the absence (Co) or the presence of EVs collected from homotypic U87 empty (E) or miR5096-loaded (M). When indicated by Msi, CLIC1 was silenced by siRNA in U87 (M). Data are means ± SD (^*^ *P*-values<0.05 *vs* control; n=10) in two independent experiments. **(F)** Contribution of CLIC1 to the branching morphogenesis in HMEC cultured in collagen ECM gel for 12 h, in the absence (Co) or presence of EVs. Histogram shows the average number of processes per cell. Control and CLIC1 siRNA HMEC are filled black and yellow, respectively. Data are means ± SD (^*^ *P*<0.05, ^**^P<0.01 *vs* Co; n=10) in two experiments. **(G)** EVs induced Ca^2+^ spikes in HMEC. Representative line scan images of cytosolic [Ca^2+^] in Fluo-4-loaded HMEC exposed to the standard solution, to EGF (10 ng/ml) and to EVs (from U87) as indicated by arrows. Space and time ordinates are displayed in the horizontal and vertical directions, respectively (scan rate 22.3 μsec/line). Amplitudes of Ca^2+^ signal are expressed as the fluorescent rapport F/F_0_ (pseudo-colors) in a tridimensional histogram (F/F_0_ *vs* space/time). In all cases, the line scan crossed both cytosol and nuclei of 4 adjacent cells, as shown in the 1024×1024 pixel panel. Note that Ca^2+^ spikes were observed at the beginning of all recordings (due to the initial cell perfusion) and were not reproduced by reapplying the standard solution.

Both U87 and HMEC expressed CLIC1 proteins, as already reported [12, 21]. Immunoblot analysis of whole cell lysates (WCL) from homotypic cultures revealed that the cell loading with miR-5096 mimic or inhibitor did not significantly change CLIC1 expression after 48h in both U87 and HMEC (Figure 1A). This is in agreement with the absence of miR-5096 effect on CLIC1 mRNA expression (not shown) and predictions from bioinformatics tools which failed to identify any target site for miR-5096 in CLIC1 gene and mRNA. However, the endothelial CLIC1 level was increased after 24h-exposure of HMEC to U87-conditioned media (Figure 1B). We next separated EVs from the effluent (soluble fraction) of culture media as described previously [18]. In all cases, EVs and effluents were adjusted to the same number of U87 (i.e. 4 × 10^6^ cells), then applied to homotypic HMEC cultures for 24h. Cell exposure to EVs released from miR-5096-loaded U87 significantly increased CLIC1 levels in HMEC, while the effluent (EVs-free) did not (Figure 1B). The immunoblot analysis of EVs showed an enrichment in the exosome specific protein tsg101 (tumor susceptibility gene 101) [8, 18] (Figure 1C). Clearly, EVs contained CLIC1 proteins and their level seemed to be higher in EVs from miR-loaded U87 than from empty-loaded U87. A possible explanation might be that miR5096 induced an increase in EVs release [18], rather than a significant increase in CLIC1 vesicular content. To confirm the transfer of CLIC1 to HMEC, endogenous CLIC1 was silenced by using *si*RNA in a series of experiments (i.e. relative OD of 0.406±0.061 and 0.015±0.007, respectively before and after CLIC1 silencing; P<0.05, n=3). As shown in Figure 1C, both cellular (WCL) and vesicular (EVs lysates) CLIC1 contents were suppressed in HMEC by CLIC1 *si*RNA. The CLIC1 immuno-labelling showed that CLIC1 was mostly found in perinuclear areas of control HMEC (Figure 1D). No labelling was observed after silencing CLIC1 in HMEC. After 24h of cell incubations with EVs from miR-5096 loaded U87, we detected CLIC1 in both the cytosol and the plasma membrane of control and silenced HMEC. We also overexpressed a fluorescent-tagged version of human CLIC1 in U87 and collected conditioned media after 48h (see Supplementary Figure 2). Exposure of HMEC to isolated EVs resulted to the fluorescent labelling of HMEC after 24h. Thus, the increase of CLIC1 in HMEC resulted more from a vesicular transfer of CLIC1 rather than an up-regulation of its endogenous expression in recipient HMEC. Are the transferred proteins active in the recipient cells? Using a 24-hour three-dimensional *in vitro* angiogenesis assay, we explored the ability of vesicular CLIC1 to induce endothelial spheroid sprouting [22]. As shown in Figure 1E, EVs stimulated HMEC sprouting even more when collected from miR-loaded U87 (M) (see also Supplementary Figure 1A). This effect was partially prevented by silencing CLIC1 in U87 (Msi). To confirm the contribution of vesicular CLIC1 to angiogenesis, EVs were also tested on branching morphogenesis *in vitro* [22]. HMEC control and CLIC1 silenced were plated in ECM gel and exposed or not (Co) to EVs collected from the same number of U87. Quantification of average number of processes per cell was performed after 12h (Figure 1F; see also Supplementary Figure 1B). Silencing CLIC1 in HMEC decreased the branching as expected for its contribution to *in vitro* angiogenesis [12]. Exposure to EVs stimulated the branching even more when collected from miR-5096-loaded U87 (M). This effect was partially prevented by silencing CLIC1 in secretory U87 (Msi). Thus, the vesicular CLIC1 was active in recipient HMEC and contributed to early steps of *in vitro* angiogenesis [12].

### EVs-mediated CLIC1 transfer to HMEC requires TRPM7-dependent Ca^2+^ signaling

**Figure 2:**
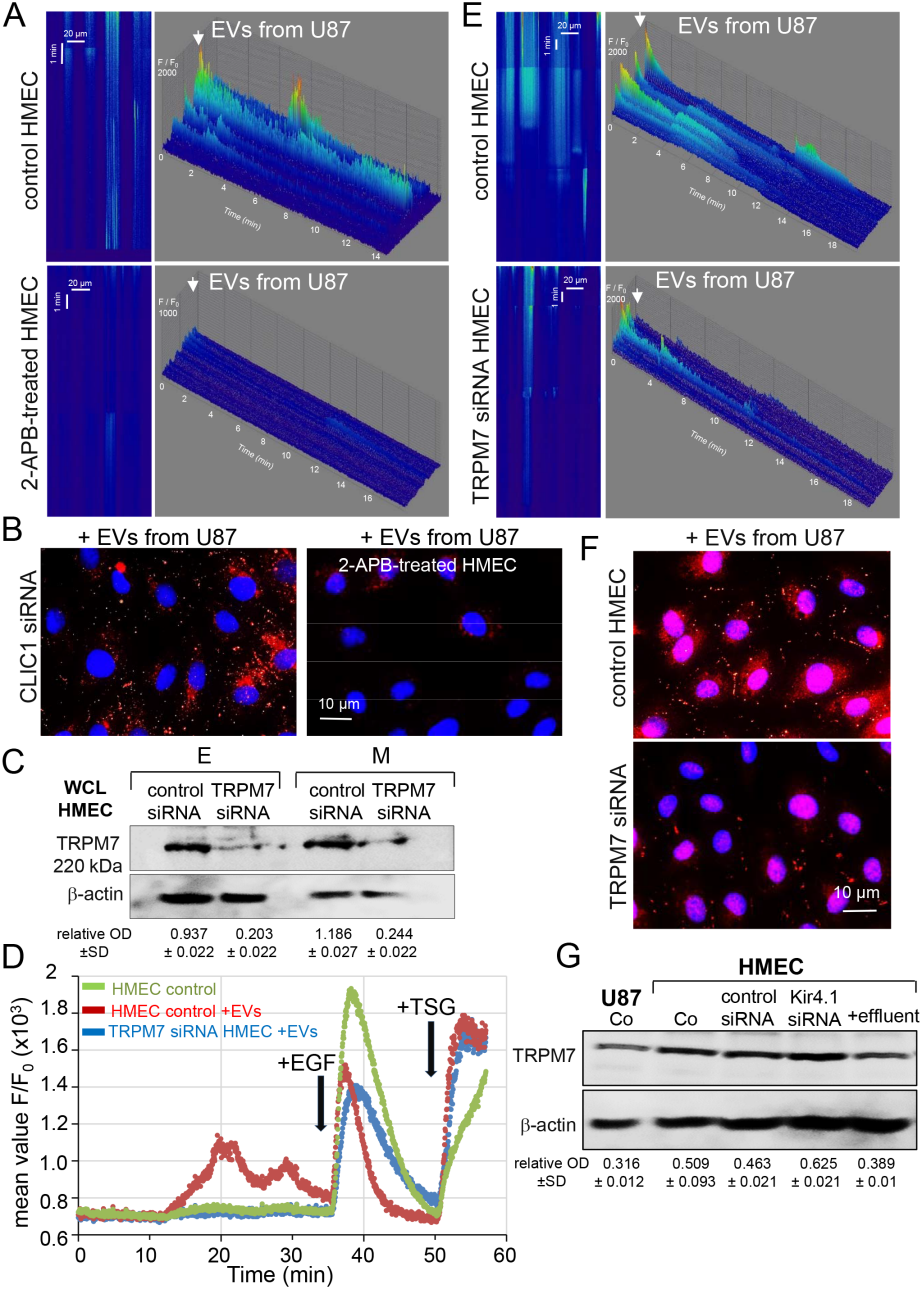
The vesicular transfer of CLIC1 requires cytosolic Ca^2+^ increases in HMEC **(A)** Line scan images of cytosolic [Ca^2+^] increases in Fluo-4 loaded HMEC exposed to the same amount of EVs collected from the same homotypic U87 culture. HMEC were pre-treated with 2-APB (50 μM; lower panel). Tridimensional histogram (F/F_0_ *vs* space/time) are representative of 3 experiments. **(B)** Transfer of vesicular CLIC1 was blocked by 2-APB (50 μM). HMEC silenced by siRNA CLIC1 were exposed to EVs from U87 (n = 3). After 24h of incubation with EVs, HMEC were stained for CLIC1 (red) and nuclei (blue). **(C)** Expression of TRPM7 in HMEC. Homotypic HMEC were loaded (M) or not (E) with miR-5096 upon transfection of control siRNA or siRNA targeting TRPM7. Numbers indicate mean OD values of TRPM7 related to β-actin (± SD; n = 2; 80μg proteins/lane). **(D)** Spatially average Ca^2+^ profile showing the dynamic change of Ca^2+^ signals with time and induced by EVs (applied at the beginning of the records) then EGF (10 ng/ml) applied at the time indicated by arrow. Cytosolic Ca^2+^ store depletion was performed by the addition of thapsigargin (TSG, 5μM) at the end of recordings. Values are means of fluorescent ratio F/F_0_ ± SD; n = 3. **(E)** Silencing TRPM7 in HMEC reduced the Ca^2+^ signal induced by EVs collected from homotypic U87 for 48h. **(F)** Control and silenced TRPM7 HMEC were exposed to EVs and stained for CLIC1 (red) after 24 h of culture (representative of 3 experiments). **(G)** Expression of TRPM7 in homotypic HMEC upon transfection of control siRNA or siRNA targeting Kir4.1 [18]. HMEC were exposed to the effluent (soluble fraction) from homotypic U87. Numbers indicate mean OD values of TRPM7 related to β-actin (± SD; n = 2).

In order for EVs to elicit a signaling response from recipient cells, they can fuse with plasma membrane or are taken up via endocytosis or attach to the cell surface [23] (see Supplementary Figure 2). Since endocytosis, receptor internalization and trafficking are regulated by cytosolic Ca^2+^ level [24, 25], we determined whether the endothelial uptake of vesicular CLIC1 was associated with cytosolic Ca^2+^ fluctuations. Spatio-temporal Ca^2+^ variations were recorded in HMEC loaded with Fluo-4 by using line scanning of confocal microscopy [26]. No Ca^2+^ spike was observed in standard external conditions (Figure 1G; upper panel). As a positive control, HMEC were exposed to epidermal growth factor (EGF), inducing a typical pattern of Ca^2+^ signal [27], i.e. a rapid increase in cytosolic Ca^2+^ which was maintained several minutes before decaying to the resting level, followed by a second transient increase observed about 6 min later. The endothelial cell exposure to EVs from U87 elicited a similar Ca^2+^ signal although with a lower amplitude than did EGF (i.e. max F/F_0_ of 1000 ± 500 with EVs and 3000±600 with EGF; P<0.05, n=6). Of note, the lag time between successive Ca^2+^ waves was similar (6.61 ± 1.25 min; P>0.05; n=6). The well-known inhibitor of Ca^2+^ pathways, 2-APB [28–31], suppressed EVs-evoked Ca^2+^ signal (Figure 2A, lower panel) as well as the subsequently CLIC1 delivery to HMEC (Figure 2B). Indeed, incubations of 2-APB-treated HMEC with EVs for 24 h did not increase CLIC1 labelling, especially in CLIC1-silenced HMEC (Figure 2B). Among 2-APB-sensitive channels in plasma membranes, the melastatin-subfamily of TRP (TRPM7) is a non-specific divalent cation channel upregulated in GBM [29]. To estimate its contribution in both Ca^2+^ waves and CLIC1 delivery to HMEC, endogenous TRPM7 was silenced by using *si*RNA (Figure 2C, 2D). Cell exposure to EVs already produced cytosolic Ca^2+^ waves which were decreased by silencing TRPM7 (Figure 2E). Unfortunately, we could not knockdown both TRPM7 and CLIC1 since HMEC did not survive. After 24h of cell incubations with EVs, CLIC1 labeling was also decreased in cytosol or plasma membrane of TRPM7-silenced HMEC, attesting the partial contribution of TRPM7 in this process (Figure 2F). Of note, cell exposure to soluble fractions (EVs-free effluent) from U87-conditioned media did not increase TRPM7 levels in HMEC, while the miR5096 loading or Kir4.1 silencing did (Figure 2C, 2G). Thus, the vesicular transfer of CLIC1 to HMEC required Ca^2+^ signaling mediated, at least in part, by TRPM7.

## DISCUSSION

We report here that CLIC1 protein is transferred via EVs from the GBM cell line U87 to microvascular endothelial cells where it remains active, i.e. induces endothelial sprouting. When applied onto HMEC, EVs elicit Ca^2+^ “spikes” which can be prevented by 2-APB. Although 2-APB inhibits numerous channels including IP_3_ receptors [28], store-operated Ca^2+^ channels [30] and TRP channels [29, 31], silencing TRPM7 prevents Ca^2+^ spikes and the subsequent CLIC1 uptake by HMEC. Altogether, our data show that CLIC1 secreted by cancer cells via extracellular vesicles, modulates the activity of neighboring endothelial cells in a TRPM7 dependent manner, promoting tumor angiogenesis.

We previously reported that miR-5096 favors its own transfer from U87 to HMEC via an increased release of EVs, two days after its loading in U87 [18]. Here, we show that EVs also contain CLIC1 and mostly ensure its transfer to HMEC (Figure 1; see also Supplementary Figure 2). EVs are composed of an array of proteins, nucleic acids, lipids, and other metabolites that reflect the cell of origin [4]. We report that EVs, secreted by the same number of U87, produce a greater increase in active CLIC1 in the recipient HMEC when the donor U87 are previously loaded with miR-5096. The most likely explanation is that miR-5096 induces an increase in EVs release [18], rather than a significant increase in CLIC1 vesicular content. How miR-5096 exerts such an effect is currently unknown and not explored in our study. Nevertheless, evidence increasingly points to a connection between lipid metabolism and cancer, characterized by an alteration in the mechanisms that regulate cholesterol homeostasis [32]. It is known that the survival of GBM cells is dependent on uptake of cholesterol [33] in which some microRNAs are the fine tuners [34, 35]. Interestingly, cholesterol promotes the conversion of CLIC1 from cytosolic to transmembrane proteins [36], thus facilitates its docking to the membranes [37, 38]. Drawing on the data above, we propose a pure speculative model where miR-5096 increases cholesterol and CLIC1 is involved in recruiting EVs, leading to an increased secretion of EVs by U87 cells (see Supplementary Figure 3).

By overexpressing fluorescent-tagged CLIC1 proteins (CLIC-OFP) in U87, we observe CLIC1-OFP inside the invadopodia (see Supplementary Figure 2B). Upon exposure to EVs, HMEC become fluorescent and change their morphology; i.e. showing invadopodia formation. Invadopodia act as multivesicular endosome docking sites and are a site of EVs release, meaning the cell's ability to form invadopodia determines their ability to release of EVs [4, 5]. In this process, CLIC1 would contribute to the formation of invadopodia in endothelial and tumor cells, by inducing integrin-mediated actomyosin dynamic [15]. Changes in CLIC1 location from cytosolic to transmembrane proteins are associated with malignant transformation [15]. Our study does not allow to distinguish the two forms of CLIC1 (i.e. soluble form and membrane-inserted chloride conducting pore). However, immuno-labeling of CLIC1 confirms its previously described nuclear location in steady HMEC [9]. Following exposure to EVs, CLIC1 is also detected in cytosol and weakly at the plasma membrane of HMEC within 24h. On the other hand, the endothelial sprouting in matrigel is increased by EVs within 24h. This effect is partially prevented by silencing CLIC1 in donor U87 and is not attributed to miR-5096 itself [39]. Our results are in agreement with the literature showing that a low CLIC1 expression in endothelium decreases capillary-like sprouting in matrigel [12, 14, 15]. We observe a functional difference between silencing all CLIC1 and preventing only the U87-derived CLIC1 delivery. This cannot be easily explained since EVs probably contain proangiogenic factors, miRNAs and extracellular proteases which are required by endothelial cells to proliferate, migrate, and organize into new tubular structures [4, 40]. For instance, EVs from xenografts of glioblastomas contain an oncogenic variant of the epidermal growth factor receptor (EGFRvIII), which can be transferred to endothelial cells, producing proliferation and tubulogenesis [41, 42]. A broad array of cell surface and signaling proteins is involved in tubulogenesis [43], and could explain why the perivascular invasion is more important in vascular endothelial growth factor (VEGF)-deficient glioblastoma cells and brain tumor xenografts treated with anti-VEGF blocking antibodies such as bevacizumab [44, 45].

We hypothesize that EVs bind to recipient endothelial cells (see Supplementary Figure 4). These EVs may remain at the plasma membrane [46] or may be internalized by endocytosis either mediated by clathrin [47, 48] or via caveolae and lipid rafts [49]. These mechanisms require Ca^2+^ increase at the submembrane level [50, 51]. While the precise mechanism of CLIC1 uptake and processing in HMEC remains unclear, we show for the first time that CLIC1 transfer requires an initial Ca^2+^ signaling in recipient cells within 1h. Moreover, the EVs-induced Ca^2+^ signal is suppressed in nominally Ca^2+^-free standard solution (i.e. no CaCl_2_ added; data not shown), attesting the involvement of an external Ca^2+^ entry. Among the putative Ca^2+^ entry pathways, the presence of functional TRPM7 channels is known in human endothelial cells [52–55]. By using specific *si*RNA, we identify TRPM7 as a mediator for this Ca^2+^ entry needed for CLIC1 uptake by HMEC. Of note, TRPM7 contributes to the EGF-induced Ca^2+^ signal, without affecting the Ca^2+^ content of internal Ca^2+^ stores sensitive to thapsigargin (see Supplementary Figure 1C).

High CLIC1 expression is involved in the progression of GBM and other tumors [56–58] and correlates with a poor patient outcome [13]. Our data foster the hypothesis that CLIC1 transfer to endothelial cells via EVs contributes to GBM growth by promoting capillary formation [12, 14, 15, 56]. Moreover, the pharmacological inhibition or silencing of TRPM7 inhibits adhesion or invasion in cancer cell lines [19, 59–61] as well as migration of HMEC [53]. Of note, TRPM7 expression is increased in miR-5096 loaded U87 (see Supplementary Figure 1D). Because the silencing of potassium Kir4.1 channels [18] also produces this increase, this up-regulation of TRPM7 should result more from a membrane potential variation than from a direct effect of miR-5096 on TRPM7 gene or mRNA [62]. Nevertheless, it is still not clear whether other proteins and miRNAs could be transferred via EVs to modulate channels in recipient cells [63, 64]. Further investigations are required to fully resolve the functional capabilities of EVs [65–67].

## MATERIALS AND METHODS

### Cells

Human primary microvascular HMEC (HMVEC-D; Lonza) and U87-MG cells (ATCC HTB-14) were grown in DMEM plus 10% FCS (5% CO_2_; 37°C). Cells were incubated 48 h in FCS-free media before use.

### Reagents

Monoclonal anti-Tsg101, anti-Hsc70 and anti-β-actin were purchased from Santa Cruz Biotech (Clinisciences, Fr). Monoclonal anti-CLIC1 (ab77214) and anti-TRPM7 (ab109438) were from Abcam. Fluo-4 acetomethyl (AM) ester was from Invitrogen (ThermoFisher). 2-Aminoethyl diphenylborinate (2-APB), thapsigargin (TSG) and other chemicals were from Sigma-Aldrich.

### Transfection

Cells were transfected by lipofectamine RNAiMAX according to the manufacturer's protocol (Invitrogen). Human hsa-miR-5096 mimic (mirVana TM miRNA, 4464066-MC22429) and inhibitor (4464084-MH22429) were purchased from Ambion (Invitrogen; ThermoFisher) [18]. Human TRPM7 siRNA (ID 1490) and CLIC1 siRNA (ID 145733) were purchased from (Ambion, AM51331). The sequences were: siRNA CLIC1 (5’-GAGCUUGUGUUGUGCUGAAtt-3’ and 5’-UUCAGCACAACACAAGCUCtt-3’); siRNA TRPM7 (5’-GGACCCUCACAGAUGCCUUtt-3’ and 5’-AAGG CAUCUGUGAGGGUCCtt-3’). To downregulate Kir4.1, cells were transfected with human *Kcnj10* siRNA SMARTpool (30 nM) purchased from Dharmacon (ThermoFisher), as we described previously [18]. To overexpress fluorescent CLIC1 proteins, we transfected U87 cells with the human CLIC1/NCC27 gene ORF cDNA clone expression plasmid, C-OFPSpark (HG15242-ACR, Sino Biological Inc.; purchased from Interchim, Montluçon, Fr). Cells were used after 48h.

### Immunoblotting

Cells were lysed in RIPA buffer, and Western blots were performed as previously described [18]. EVs pellets were lysed in RIPA buffer containing protease inhibitor cocktail (Roche, Indianapolis, IN) then sonicated for 10 s. Insoluble material was pelleted by centrifugation for 15 min at 14,000 g at 4°C.

### Immunofluorescence

Cells were fixed in 4% PFA and permeabilized with 0.1% Triton X-100. Images were performed using a Leica SP2 RS confocal microscope (Z-series of 0.6 μm-optical sections; 512×512 pixels).

### EVs isolation

After 48h of culture in FCS-free conditions, cell-conditioned media were collected and sequentially centrifuged at 300 g for 10 min (4°C) then at 2,000 g for 10 min to remove cell debris [18, 68]. EVs were collected by ultracentrifugation at 100,000 g for 90 min. Concentrations were adjusted to the same number of cells (i.e. corresponding to the secretion from 4 × 10^6^ cells).

### Cell sprouting assay in collagen gels

Sprouting of HMEC spheroids was performed as previously described [69]. For each gel, 8 spheroids (each containing 400 – 500 cells) were seeded into 0.7 ml collagen solution in 24-well plates (PromoCell GmbH) and incubated with the tested solutions for 24h (5% CO_2_; 37°C). The cumulative sprout length of 8 randomly selected spheroids was measured for each tested group.

### Endothelial tube formation assay in collagen gels

Control and silenced CLIC1 HMEC were plated in DMEM ECM gel, with or without EVs collected from U87, according to the manufacturer's instructions (from Cell Biolabs, Inc). After 12 hours of incubation at 37°C, 80 single cells were scored for the number of processes per cell. Each well is duplicated for each experiment, and each experiment was repeated three times.

### Calcium imaging

Spatiotemporal Ca^2+^ variations were recorded in HMEC cultured on uncoated glass then loaded with 4μM Fluo4/AM in FCS-free conditions for 40 min at 37°C [26]. Cells were bathed in the standard solution containing (in mM): 136 NaCl, 5 KCl, 1 MgCl_2_, 1.8 CaCl_2_, 0.3 NaH_2_PO_4_, 10 Glucose, and 10 HEPES (pH 7.4). Measurements were performed by a line scan crossing 3-4 cells in a 1024×1024 pixel panel using a confocal microscope (Nikon C1Si) with 100x objective (Nikon, Melville, NY). Excitation was at 488 nm and emission-selected at 500-570 nm. Line scan images in pseudo-colors were acquired at a sampling rate of 22.3 μsec per line (32 lines/sec). To compare cytosolic [Ca^2+^] fluctuations, fluorescent measurements (F) were expressed as the F/F_0_ ratio, where F_0_ refers to the basal [Ca^2+^] fluorescence at rest [70]. The signal amplitudes were shown in a tridimensional histogram (F/F_0_ as a function of time/length).

### Statistical analysis

Results are expressed as mean ± SD. A Mann-Whitney *U* test was used to compare data groups. Statistics were also made with Tanagra software using a Kruskal-Wallis 1-way ANOVA. In all cases, ^*^ *P* values < 0.05 were significant.

## SUPPLEMENTARY MATERIALS FIGURES AND TABLES


