# Supplementary material for: The vesicular transfer of CLIC1 from glioblastoma to microvascular endothelial cells requires TRPM7
Source: Oncotarget. 2018 Sep 7;9(70):33302–11. doi: 10.18632/oncotarget.26048 (PMC6161795; doi:10.18632/oncotarget.26048)
Supplement: Supplementary file 1 [file oncotarget-09-33302-s001.pdf]

The vesicular transfer of CLIC1 from glioblastoma to microvascular endothelial cells requires TRPM7

SUPPLEMENTARY MATERIALS

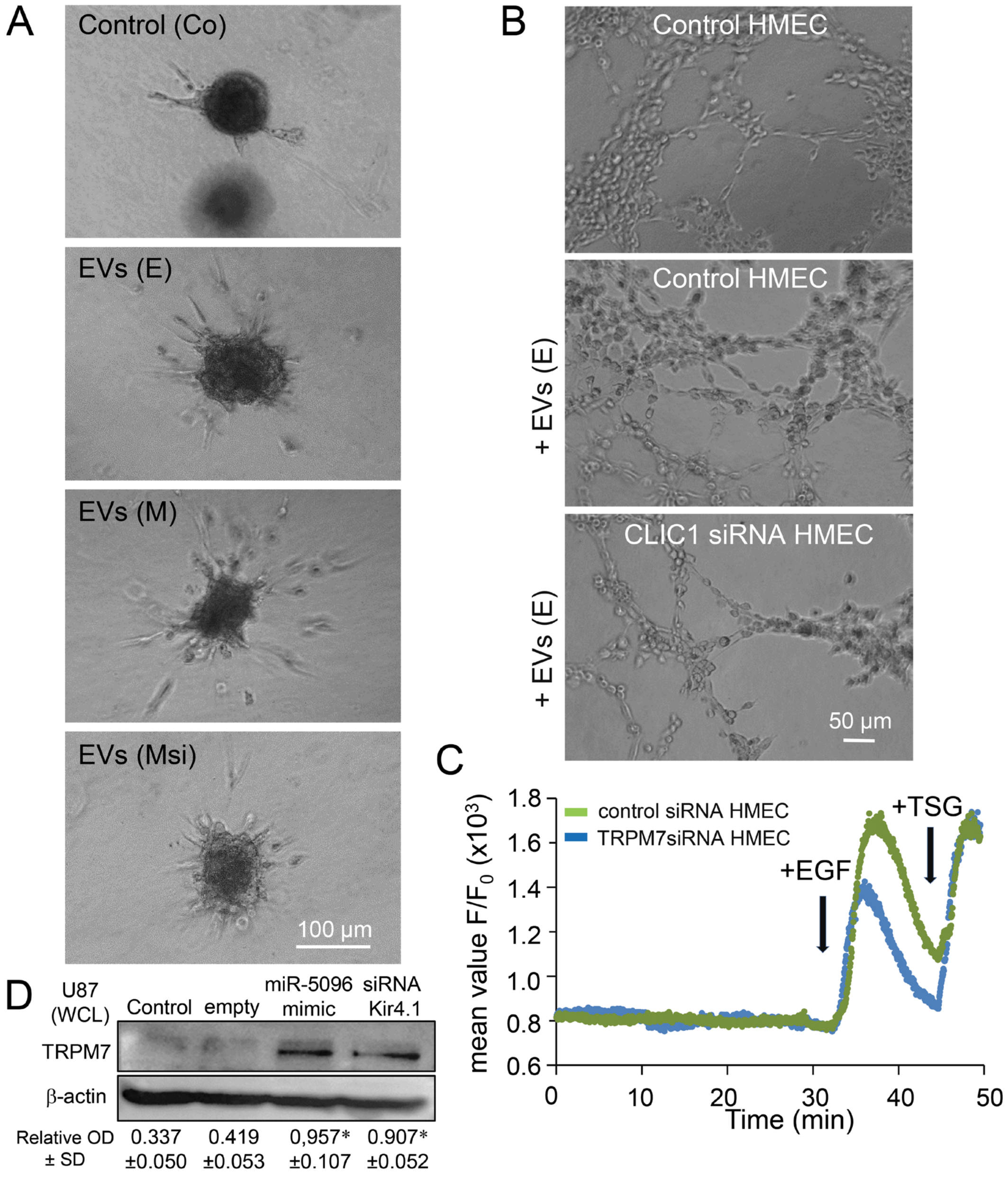

Supplementary Figure 1: (A) Representative spheroids formed from HMEC in collagen for 24 h. HMEC were exposed to EVs collected from an equal number of U87, loaded (M) or not (E) with miR-5096. CLIC1 was silenced in U87 (M) by siRNA (Msi). **(B)** EVs induced branching morphogenesis of HMEC in matrigel for 12 h. HMEC were transfected with CLIC1 siRNA. **(C)** Silencing TRPM7 in Fluo-4-loaded HMEC depressed the Ca^{2+} signal induced by EGF (10 ng/ml). The Ca^{2+} store depletion was induced by 5 μM thapsigargin (TSG). Silencing TRPM7 did not affect Ca^{2+} content of internal stores (n=3). **(D)** Western blot of TRPM7 in U87, 48 h after loading with miR5096 (M) or silencing Kir4.1 channels by siRNA [11]. Unloaded cells were labelled empty. Numbers are mean OD (normalized with β-actin) of bands relative to control (± SD; $^*P<0.05$ *vs* basal; n = 3).

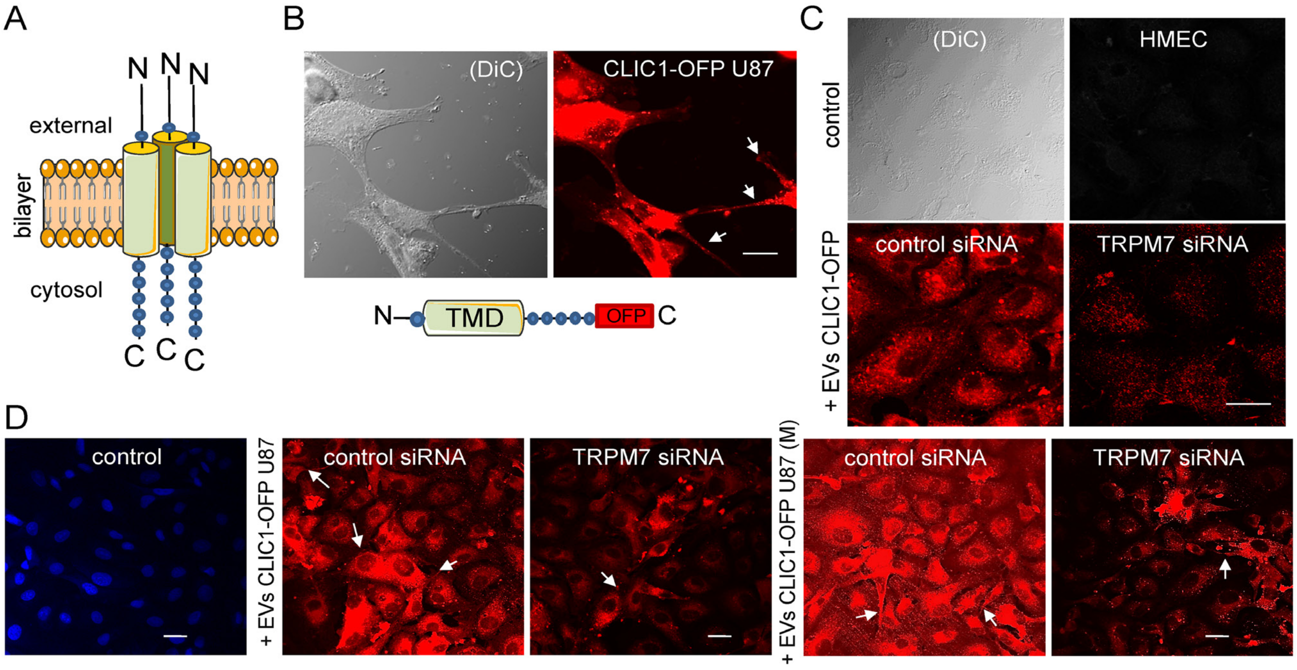

Supplementary Figure 2: (A) Membrane CLIC1 subunits modeled as a tetrameric ion channel, with the front subunit removed. **(B)** CLIC1-OFP proteins were expressed in plasmid transfected U87 (arrows indicated "invadopodia"). Human CLIC1 (cDNA encoding a C-terminal OFPSpark tag) was inserted into pCMV3 to produce a C-terminal red CLIC1 protein. View of a single subunit where « TMD » is a transmembrane domain, and the six putative cysteine residues (blue balls) are indicated. **(C)** Silencing TRPM7 prevented the vesicular transfer of CLIC1-OFP to HMEC. EVs were collected from CLIC1-OFP U87, 48 h after the plasmid transfection. Upon transfection with control siRNA or siRNA targeting TRPM7, HMEC were exposed for 24 h to EVs (representative of 3 independent experiments). **(D)** miR5096 improved the vesicular release of CLIC1. EVs were collected from the same number of CLIC1-OFP U87, loaded or not with miR-5096 (M). EVs were then applied for 24h to homotypic HMEC, transfected with control siRNA or siRNA targeting TRPM7. Note the morphological changes of red HMEC. Control HMEC stained with Dapi. Scale bar 25 µm.

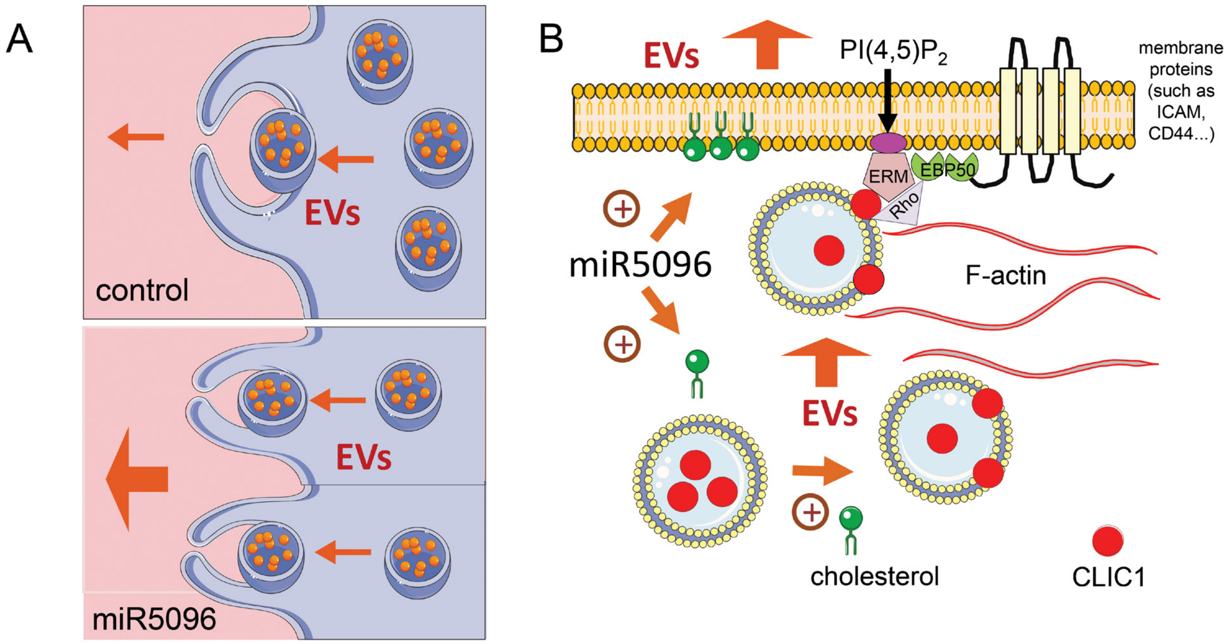

Supplementary Figure 3: Speculative model where miR-5096 increases the release of CLIC1-containing vesicles by glioblastoma cells. (A) miR-5096 increases the release of EVs without affecting the CLIC1 expression level. As reported for other miRNAs, miR-5096 could increase intracellular cholesterol levels and consequently the protein secretion. By increasing membrane cholesterol, miR-5096 would also increase the conversion of CLIC1 from the soluble to an integral membrane form. How cholesterol induces the CLIC1 membrane insertion is currently unknown. **(B)** CLIC1 is involved in recruiting vesicles. The interface between the actin cytoskeleton and plasma membrane is tightly controlled by ERM proteins: ezrin, radixin, and moesin. According to the Jiang's model [37], the vesicular CLIC1 and ERM proteins determine the fate of intracellular vesicles - to fuse or not with the plasma membrane to be secreted. The molecular mechanism by which CLIC1 (red) and ERM (pink) cooperate in this is yet unknown. In the quiescent state, vesicular CLIC1 lies in the perinuclear region. Upon activation by PI(4,5)P2, the ERM proteins function as a cross-linker by binding to cytoplasmic extensions of membrane proteins (yellow) and scaffolding proteins such as EBP50 (green). RhoGTP (purple) also binds ERM, helping in its activation via a kinase. It is associated with plasma membrane regions containing densely packed actin filaments (F-actin), in cellular structures such as invadopodia. The vesicle-bound CLIC1 are transported to the site of activation. Thus, by increasing cholesterol levels, miR-5096 would increase the insertion of CLIC1 into EVs, their transport to the plasma membrane (i.e., invadopodia) and their secretion. Further studies are necessary to validate the effect of miR-5096 on cholesterol biosynthesis in glioblastoma cells.

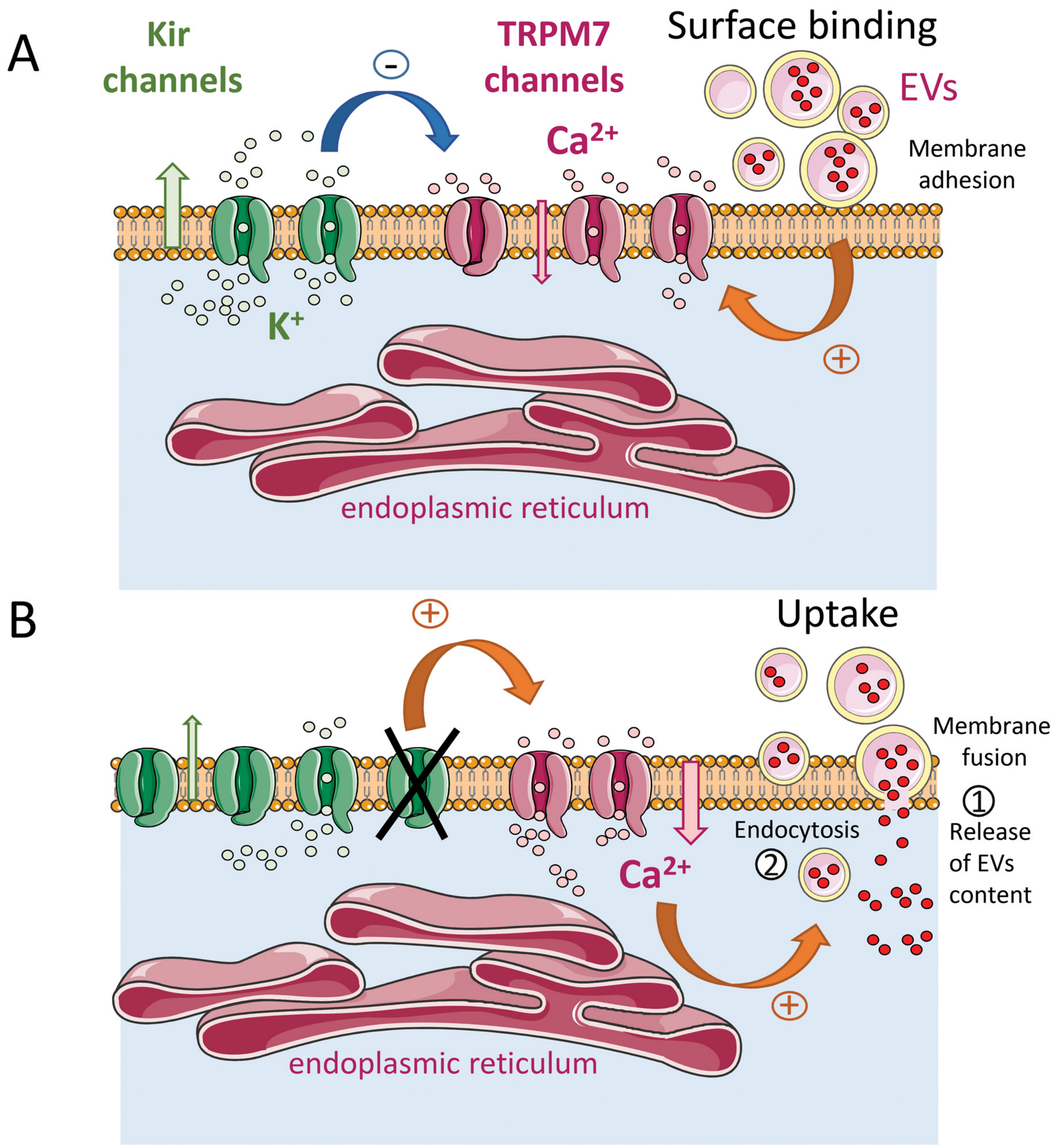

Supplementary Figure 4: Hypothetical fate of EVs in recipient cells. (A) In steady conditions, potassium Kir channels exert a negative-feedback (blue arrow) through the membrane hyperpolarization (K^+ efflux; green arrow) to decrease external Ca^{2+} entry (rose arrow). The membrane cell adhesion of EVs exerts a positive feedback (orange arrow) through the opening of TRPM7 channels to increase external Ca^{2+} entry. Several mediators of these effects are known, including tetraspanins, integrins, lectins, heparan sulfate, extracellular matrix components… **(B)** The consecutive increase in cytosolic [Ca^{2+}] is amplified by a positive-feedback contribution (orange arrow) of Ca^{2+} release from ER citernae, and the blockage of Kir channels. Depending on this Ca^{2+} increase, EVs can undergo various fate: (1) they can remain bound to the surface and initiate signaling pathways (leading to release their intraluminal contents into the cytosol); or (2) they can be internalized in multivesicular endosomes and recycle their contents to fuel the metabolism of the recipient cell. The process (1) is currently poorly understood but of a major importance for delivery of CLIC1 and microRNA.
